# Report of the Central Veterinary School at Munich, Bavaria, for the Years 1883–84

**Published:** 1885-10

**Authors:** 


### REPORT OF THE CENTRAL VETERINARY SCHOOL, AT MUNICH, BAVARIA, FOR THE YEAR 1883-4.

Owing to the death of Prof. Franck, the direction of this school has been placed in the hands of Prof. Karl Hahn.

While the Munich school is not so extensive in lands and buildings as many others in Europe, the proof that it is second to none of them in the earnestness and activity of its faculty is abundantly demonstrated by the following scientific and literary contributions during the year in question.

In this regard it bears a remarkable comparison with the utter stagnation that exists in the most completely fitted school in Europe, that at Berlin.

From Dr. Bonnet we have: A contribution to the embryology of ruminants.

From Prof. Feser: 1st—The action and employment of physostigmim sulfuricum in horses and cattle; 2d—The relation of the centifugal method to the dairy; 3d—A report upon the cattle at the exhibition at Hamburg, 1883; 4th—Studies upon the cattle of Bavaria; 5th—The better use of the cow milk in the Algan district.

Prof. Horz: A text-book upon agricultural seeds and other contributions of the same kind, as well as those of a bacteriological nature.

Dr. Tappeiner gives four valuable contributions of a chemical character, especially one on the chemical diagnostic examination of the patient.

From Dr. Kitt we have: 1st—A contribution to the early history of the horse; 2d—Studies upon the formation of the skull among Bavarian cattle—a morphological character; 3d

—The influences of breeding upon bos brachyceros; 4th—The same, extended; 5th—Numerous short contributions to bacteriology, all founded upon original studies.

The attendance during the winter session of 1883-4 was 112 students; summer, 96; number of successful graduates, 13. The pathological museum was enriched by the addition of 139 new specimens.

Whole number of animals treated in clinic, 552; number of forensic examinations for soundness, 90; number of animals with contagious diseases brought to hospital, 6; number of animals treated in free clinic, 869.

Dr. Kitt gives a very interesting continuation of the studies begun by Schultz and Loeffler, of Berlin, upon the bacillus of glanders. The material was taken from a thoroughly intact Farcy-bud, after the hair had been carefully shaved off, and the skin thoroughly washed with a solution of mercuric chlorid. The bud was then opened with knives that had been previously subjected to a red heat. The contents of the bud was at once distributed in the smallest quantities possible in isolated spots over the surface of sterilized potatoes and upon blood serum. Noduli from the lungs were also used under similar precautions. The cultivations were made in the thermostat at a temperature of 37.8° C., and in the ordinary temperature of the laboratory. Notwithstanding the care taken in procuring the material some foreign germs were seen to have developed upon the potatoes inoculated with pulmonary material. Upon the others the cultivations remained pure. On the blood serum one could see—third or fouth day—isolated yellow points, which soon increased in size, that both examination and experiment proved to be true glanders bacilli. Cultivations on potatoes have a pale varnish yellow color. The bacilli of glanders are somewhat smaller than those of tuberculosis, having a length of .0007—.0024 mm., but are a little thicker. They color easily in methyl-violet. They probably develope spores. The inoculation of rabbits with pure cultivations of these bacilli gave unmistakable indications of nasal and pulmonary glanders on microscopical examination. The same author also describes a continued series of experiments with the germ of fowl cholera which, according to him, is coccus, occasionally having the form of a diplo, or double coccus.

Pure cultivations of this germ upon flesh-water gelatine, and solidified blood serum appear as a pale, transparent white coating or mycoderm upon the surface of the medium ; they do not cause fluidification of the medium. Some of the cultivations of the first generation were polluted by the presence of an extremely delicate bacillus. These cultures had a very offensive odor which was not present in those of a pure character.

Numerous experiments were made upon fowls as well as upon animals. In the latter abscesses developed, but a fatal termination did not always follow, except in rabbits and small animals.

The cultivations retained their virulence for over four months. These cocci were found to pass through the placental circulation into the fœtus in healthy guinea pigs, as well as into the yolk in the hen eggs. Almost all the animals that perished from the experiments were fed to three young dogs without causing any disturbance.

